# Single-stage versus two-stage resection for large anterior midline skull base meningiomas with bihemispheric peritumoral edema

**DOI:** 10.1038/s41598-025-92516-5

**Published:** 2025-03-07

**Authors:** Lina-Elisabeth Qasem, Ali Al-Hilou, Jan Oros, Katharina J. Weber, Fee Keil, Daniel Jussen, Vincent Prinz, Volker Seifert, Peter Baumgarten, Gerhard Marquardt, Marcus Czabanka

**Affiliations:** 1 Center for Neurology and Neurosurgery, Department of Neurosurgery, Goethe University Frankfurt, University Hospital, Frankfurt am Main, Germany; 2 Neurological Institute (Edinger Institute), Department of Neuropathology, Goethe University Frankfurt, University Hospital, Frankfurt am Main, Germany; 3 Goethe University Frankfurt, Frankfurt Cancer Institute (FCI), Frankfurt am Main, Germany; 4 German Cancer Consortium (DKTK), Partner Site Frankfurt, and German Cancer Research Center (DKFZ), Heidelberg, Germany; 5 Goethe University Frankfurt, University Cancer Center (UCT), Frankfurt am Main, Germany; 6 Department of Neuroradiology, Goethe University Frankfurt, University Hospital, Frankfurt am Main, Germany; 7 Department of Neurosurgery, University Hospital Jena, Jena, Germany; 8 Department of Neurological Surgery, University Hospital Frankfurt am Main, Schleusenweg 2-16, 60528 Frankfurt am Main, Germany

**Keywords:** Anterior skull base meningiomas, Meningioma, Olfactory groove meningiomas, Peritumoral edema, Skull base surgery, Complications, Brain, Outcomes research, CNS cancer, Surgical oncology

## Abstract

Resection of large anterior midline skull base meningiomas with extensive peritumoral edema poses high risks due to postoperative edema decompensation leading to increased intracranial pressure. Initial craniectomy prevents intracranial pressure decompensation but requires secondary cranioplasty. This study compares single-stage osteoplastic craniotomy with tumor resection to a two-stage approach using bifrontal craniectomy, tumor resection and subsequent cranioplasty after edema recovery in a second surgical step. Patients with large anterior midline skull base meningiomas (> 50 mm) and extensive peritumoral edema were included. Group 1 underwent single-stage resection (2002–2016), while Group 2 had a two-stage approach (2012–2022). The primary outcome was the Karnofsky Performance Scale (KPS) at three months post-surgery. Secondary outcomes included preoperative KPS, KPS at discharge and last follow-up, ICU stay, hospital stay length and complication rates. A total of 25 patients were analyzed (Group 1: *n* = 9; Group 2: *n* = 16). Group 2 demonstrated significantly improved KPS at three months postoperatively (median KPS 70% vs. 50%; *p* = 0.0204) with a non-significant reduction in ICU stay (10 vs. 6.5 days; *p* = 0.3284). Although no significant differences were observed in KPS at discharge (Group 1: KPS 30% vs. Group 2: KPS 50%; *p* = 0.1829) or last follow-up (Group 1: KPS 60% vs. Group 2: KPS 80%; *p* = 0.1630), Group 2 patients required fewer postoperative interventions for complications unrelated to cranioplasty. Overall complication rates were comparable in both groups (Group 1: 67% vs. Group 2: 56%; *p* = 0.6274). Two-stage resection of large anterior midline skull base meningiomas with extensive edema provides superior clinical outcomes at three months postoperatively without increasing overall complication rates. These findings support the use of a two-stage surgical strategy for highly selected patients. However, further multicenter studies are warranted to validate these results in larger cohorts.

## Introduction

**Fig. 1:**
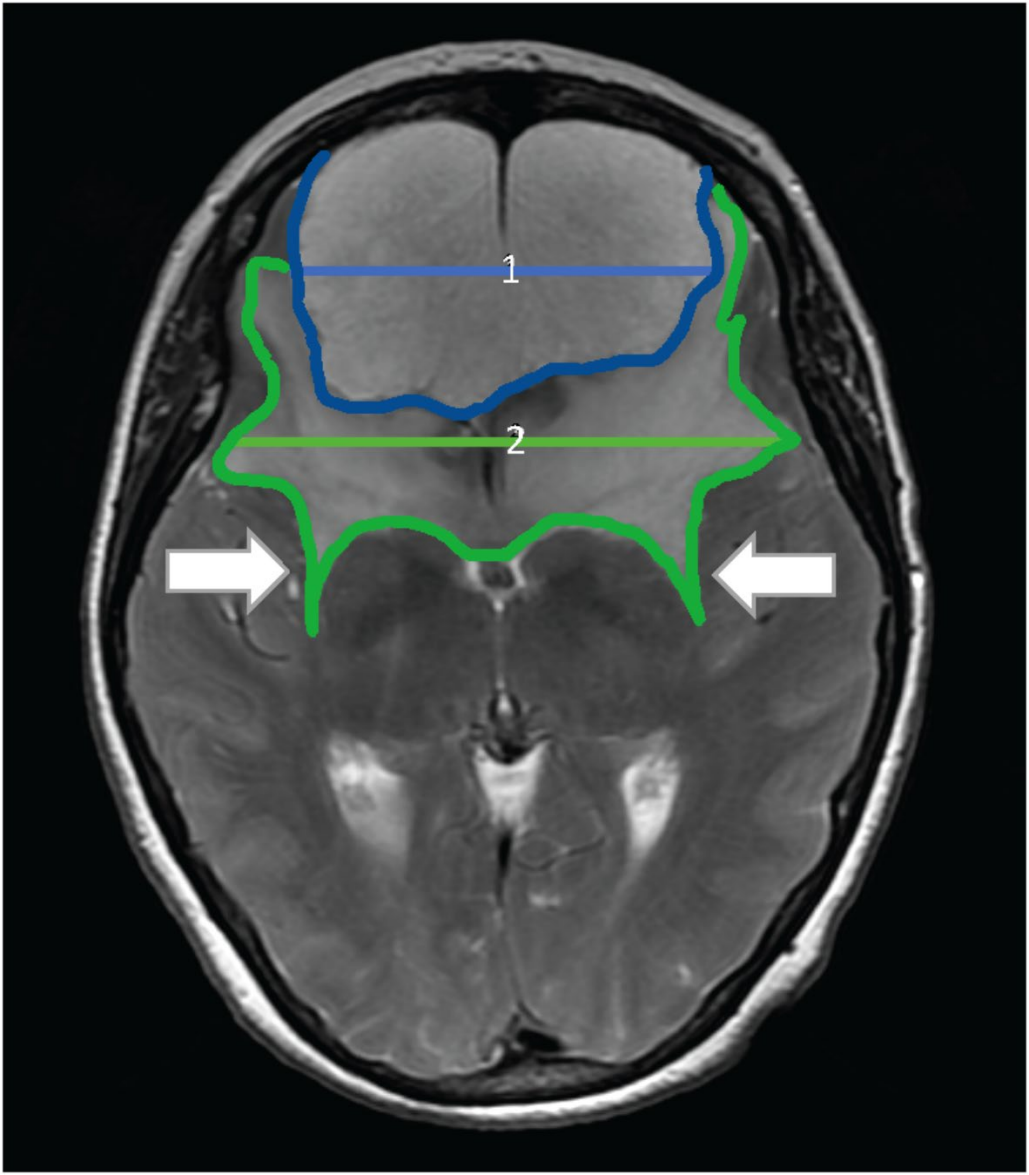
Olfactory groove meningioma with extensive peritumoral edema. T2-weighted MRI sequence of a patient with a large (maximum tumor diameter > 50 mm; 1 = 71 mm; highligthed in blue) olfactory groove meningioma with extensive bihemispheric, peritumoral edema (maximum edema diameter > maximum tumor diameter; 2 = 98 mm; highlighted in green). The white arrow marks the so-called “sabre-tooth” sign^6^, which describes a specific pattern of severe cerebral edema extending posteriorly within the external capsule.

The overlapping symptoms of large anterior midline skull base meningiomas may occur late in the clinical course due to the lack of functional cerebral regions adjacent to the tumor and the patient’s tendency to ignore mild and subtle symptoms. As a result, these tumors are among the largest tumors seen by neurosurgeons^1^. Radiographically, these slow-growing and homogeneously enhancing tumors often present with large peritumoral edema (see Fig. 1), leading to increased symptoms and surgical challenges^2–5^. Surgery, with the goal of complete tumor removal, is the treatment of choice, despite the fact that large tumor size and extensive peritumoral edema may complicate surgical management^6^. Consequently, resection of anterior midline skull base meningiomas is associated with high morbidity and mortality of up to 5%, due to neurovascular conflicts involving the anterior cerebral arteries and the risk of postoperative decompensation of peritumoral edema leading to increased intracranial pressure^6–9^. In these patients, we may see dramatic clinical deterioration, followed by reintubation, decompressive craniectomy and even death due to uncontrollable intracranial pressure. Nakamura et al.^8^ described three cases of death due to extensive postoperative brain edema in their series of 82 patients. The perioperative complication rate is reported to be high up to 39%^1,6–8,10,11^. In a previous study, we identified a subgroup of anterior midline skull base meningiomas that we termed “high-risk”, characterized by significantly worse outcomes and an increased complication rate. This subgroup includes patients with large meningiomas (> 50 mm) and bihemispheric extensive peritumoral edema.

To reduce postoperative morbidity and mortality, a two-stage surgical procedure has been introduced at our department to control postoperative decompensation of intracranial pressure by performing tumor resection via a craniectomy followed by delayed cranioplasty after regression of brain edema^12^. This strategy promises superior control of intracranial pressure to avoid subsequent complications, but requires secondary cranioplasty, which in turn implies an additional risk profile for the patient^5,9–11,13–15^. Therefore, the aim of the current study was to compare a single-stage resection strategy (i.e., osteoplastic craniotomy followed by microsurgical tumor resection) with a two-stage approach using bifrontal craniectomy, tumor resection, and subsequent cranioplasty after edema decline in terms of clinical outcome and complication profile.

## Materials and methods

### Study design

Patients with anterior midline skull base meningiomas greater than 50 mm in diameter and bihemispheric peritumoral edema greater than the tumor diameter^16,17^ were included in the study (see Fig. 1). Group 1 patients were treated with single-stage tumor resection by osteoplastic bifrontal craniotomy and served as the historical control. To adjust the groups in terms of number of patients, we included more patients treated with single-stage resection dating back to 2002. Group 2 patients were treated between 2012 and 2022 with a two-stage approach of bifrontal craniectomy, tumor resection and subsequent cranioplasty. The cohorts were matched based on age and gender to minimize potential confounding factors. Using a caliper matching approach with a tolerance of ± 5 years for age, we ensured that each participant in Group 2 had a corresponding participant in Group 1. The final analysis included participants in each cohort, with no significant differences observed in baseline characteristics.

Approval from the Ethics Committee of the Goethe University Frankfurt, University Hospital, Frankfurt am Main, Germany was obtained prior to the study. This research was conducted in accordance with the Declaration of Helsinki and all relevant guidelines and regulations. As the study involves retrospective data analysis, a waiver of informed consent was granted by the Institutional Review Board (IRB) of Goethe University Frankfurt am Main, Germany, where broad consent covers retrospective data analysis. All patients provided broad consent, consequently no additional patient consent was required for the analysis. Routine surgical consent was obtained prior to surgery.

### Outcome parameters

The primary outcome measure was the Karnofsky Performance Scale (KPS) score at three months after surgery. Preoperative KPS, KPS at discharge and at last follow-up were analyzed as secondary parameters, as well as length of stay in the intensive care unit (ICU), elective mechanical ventilation, length of hospital stay and complication rate. All data were retrieved from the hospital’s record system. The KPS at three months post-surgery and at the last follow-up was assessed during routine follow-up appointments in our outpatient clinic.

Complications were divided into early and late postoperative complications, with early complications occurring during the initial hospital stay and late postoperative complications, occurring after patient discharge. Early complications were further subdivided into major complications, requiring surgical treatment or intervention, and minor complications, requiring medical treatment or observation in the ICU. Partial data from both cohorts have been published previously^12^.

### Surgical technique

**Fig. 2:**
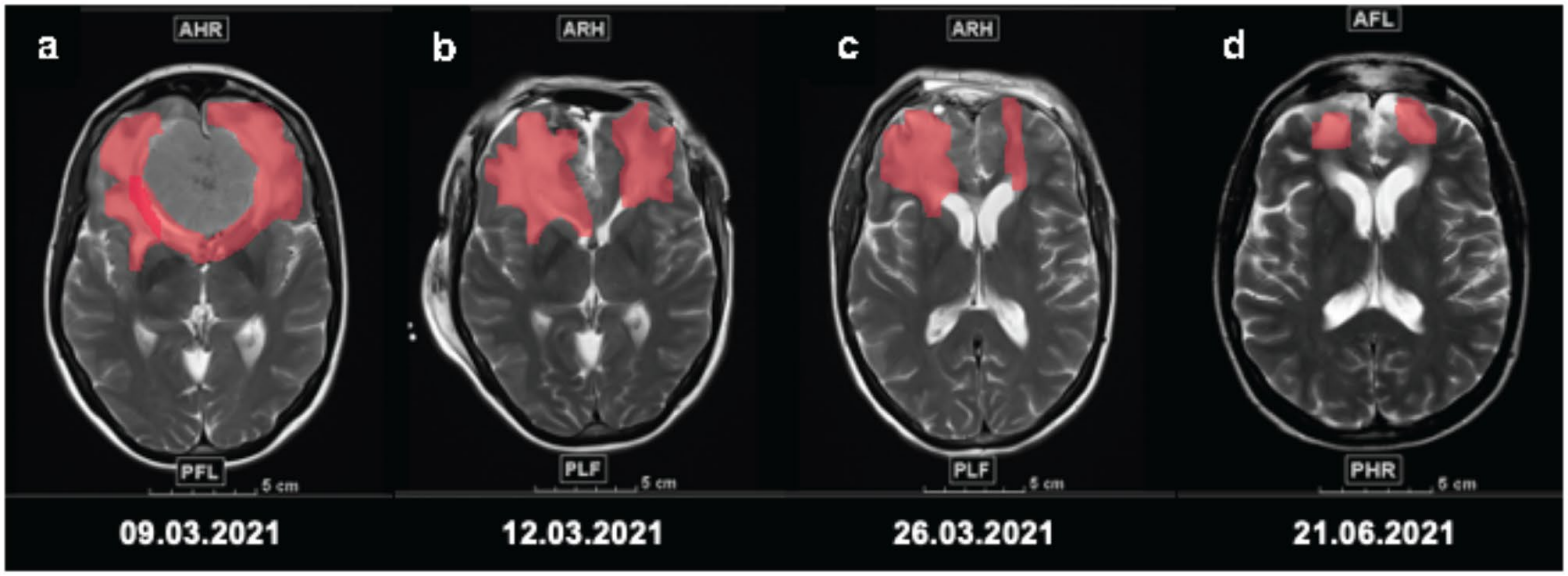
Progression of peritumoral edema. Axial T2-weighted MRI scans of a patient with a large olfactory groove meningioma with extensive peritumoral edema. Panels a-d illustrate the progression of edema over time. The edema is highlighted in red to enhance visualization. Panel a shows the expansion of edema preoperatively. Two-stage resection was performed on March 11, 2021. Panel b shows edema on the first postoperative day. Autologous bone grafting was performed on March 29, 2021. The patient recovered well from both surgeries.

Single-stage surgery was defined as conventional tumor removal via bifrontal craniotomy and interhemispheric approach. Two-stage surgery was defined as tumor removal via a subfrontal or interhemispheric approach using a bifrontal craniectomy. Bifrontal craniectomy was performed without opening the frontal sinus to avoid sinus-associated complications. The bone flap was not reinserted to avoid complications associated with progressive edema and neurological deterioration of the patient. The dura was left open and the bone graft was stored in a deep freezer at -80 °C. In a second surgical step, autologous bone grafting was performed after cerebral edema had resolved and patient recovery. The time from resection to cranioplasty ranged from several days to weeks, depending on the clinical status and postoperative complications. In patients exhibiting stable clinical status without complications, cranioplasty was performed following confirmation of edema decline on scheduled postoperative imaging. Conversely, for patients with compromised clinical status, the procedure was deferred until clinical improvement was observed and imaging confirmed edema decline. Figure 2A-D provides a radiographic illustration of the progression of edema over time in a two-stage approach.

### Statistical analysis

Data were analyzed using GraphPad Prism 9.5 (GraphPad software version 9.5.1; 528; San Diego, CA, USA). Descriptive statistics were performed for all variables, with continuous data presented as means with standard deviations (SDs) or medians with ranges, as appropriate. The normal distribution of variables was assessed using the Kolmogorov-Smirnov test. Comparisons of nonparametric data between two subgroups groups were evaluated using the Mann-Whitney U test, while the unpaired t-test was applied for parametric data. Differences between three subgroups for nonparametric data were assessed for significance using the Kruskal-Wallis test, followed by post-hoc analysis using the Mann-Whitney U test, and ANOVA analysis for parametric data, followed by the unpaired t-test. 95% confidence intervals (CIs) were calculated for key outcome measures and for non-significant findings. P values < 0.05 were considered statistically significant.

## Results

Twenty-five patients were included in the analysis. Nine patients who underwent single-stage surgery were included in Group 1. Sixteen patients underwent surgery as part of a two-stage procedure and were included in Group 2.

### Baseline patient characteristics

Group 1 consisted of four female and five male patients with a median age of 56 years (range 44–79). Group 2 consisted of nine female and seven male patients for a total of 16 patients with a median age of 50 (range 43–83). Median preoperative KPS in Group 1 was 75% (range 60–90%) and 80% (range 40–100%) in Group 2 (*p* = 0.8160). Most patients presented with cognitive dysfunction (*n* = 13; 52%), followed by anosmia (*n* = 8; 32%), visual impairment (*n* = 6; 24%), and seizures (*n* = 6; 24%). Most patients presented with more than one symptom. The mean follow-up time of Group 1 was 67 (±65) months and 26 (±33) months in Group 2.

### Radiographic and surgical parameters

The mean tumor diameter was 62 (±8) mm in Group 1 and 62 (±10) mm in Group 2 (*p* = 0.7920). The mean tumor volume was 110 (±124) cm^3^ for Group 1 and 132 (±188) cm^3^ for Group 2 (*p* = 0.6442). The mean diameter of the peritumoral edema was 89 (±25) mm in Group 1 and 124 (±60) mm in Group 2 (*p* = 0.2735) and the mean edema volume was 101.8 (± 67) cm^3^ for Group 1 and 124 (± 60) cm^3^ for Group 2 (*p* = 0.5320).

All patients in Groups 1 and 2 underwent bifrontal craniotomy or bifrontal craniectomy. In Group 1, all patients (*n* = 9; 100%) were treated using an interhemispheric approach. In Group 2, 13 patients (81%) underwent an interhemispheric approach, while three patients (19%) underwent a subfrontal approach.

Microscopic complete resection was achieved in 9/9 cases in group 1 (100%). However, in one case, a tumor remnant was detected on the three months follow-up MRI (11%). Tumor recurrence occurred in two cases, at 70 months and 189 months (22%). Both cases were managed with annual follow-up MRIs, as the recurrent tumors were not space-occupying, and therefore, reoperation was not deemed necessary. In Group 2, microscopic complete resection was achieved in 12/16 cases (75%; *p* = 0.2601). In 3 cases (19%), a residual tumor was detected on the three months follow-up MRI. The recurrence rate in Group 2 was 19%, with three cases of recurrence occurring at three, 38, and 47 months postoperatively. Two cases were managed with follow-up MRIs, while one case required reoperation to decompress the optic nerve.

No significant differences were observed between the two surgical groups regarding demographic characteristics, localization, clinical presentation, radiographic parameters, complete resection rate, and recurrence rate. However, differences in follow-up durations can significantly influence recurrence rates, as longer follow-up periods may capture more late-onset recurrences. All baseline patient characteristics including radiographic and surgical parameters are summarized in Table 1.

**Table 1:** Baseline patient characteristics.

	single-stage surgery	two-stage surgery	*p*-value
total n (% of total)	**9**	**16**	
newly diagnosed	9 (100%)	16 (100%)	
recurrence	0	0	
age in years median (range)	56 (44–79)	50 (43–83)	0.6665
gender n (% of total)			
female	4 (44%)	9 (56%)	0.5893
male	5 (56%)	7 (44%)	0.5893
localization n (% of total)			
olfactory groove	9 (100%)	15 (94%)	0.4650
tuberculum sellae	0	0	
planum sphenoidale	0	1 (6%)	0.4650
CNS WHO grade n (% of total)			
1	5 (56%)	7 (44%)	0.5893
2	4 (44%)	8 (50%)	0.8000
3	0	1 (6%)	0.4650
preoperative KPS in % median (range)	75 (60–90)	80 (40–100)	0.8160
follow-up (months) mean (standard deviation)	67 (±65)	26 (±33)	0.0561
clinical presentation n (% of total)			
cognitive disturbance	4 (44%)	9 (56%)	0.5893
anosmia	6 (67%)	7 (44%)	0.2903
seizure	2 (22%)	4 (25%)	0.8822
visual impairment	2 (22%)	4 (25%)	0.8822
headache	2 (22%)	3 (19%)	0.8432
depression	1 (11%)	1 (6%)	0.1111
aphasia	0	1 (6%)	0.4650
urine incontinence	1 (11%)	1 (6%)	0.111
maximum tumor diameter in mm mean (standard deviation)	62 (±8)	62 (±10)	0.7920
tumor volume in cm^3^ mean (standard deviation)	110 (±124)	132 (±188)	0.6442
maximum edema diameter in mm mean (standard deviation)	89 (±25)	124 (±80)	0.2735
edema volume in cm^3^ mean (standard deviation)	102 (±67)	124 (±60)	0.5320
complete microscopic resection in %	100%	75%	0.1102
complete radiographic resection in %	89%	81%	0.7920
recurrence in %	22%	19%	0.7999

Abbreviation: central nervous system (CNS); Karnofsky Performance Scale (KPS); World Health Organization (WHO).

### Outcome parameters

**Fig. 3:**
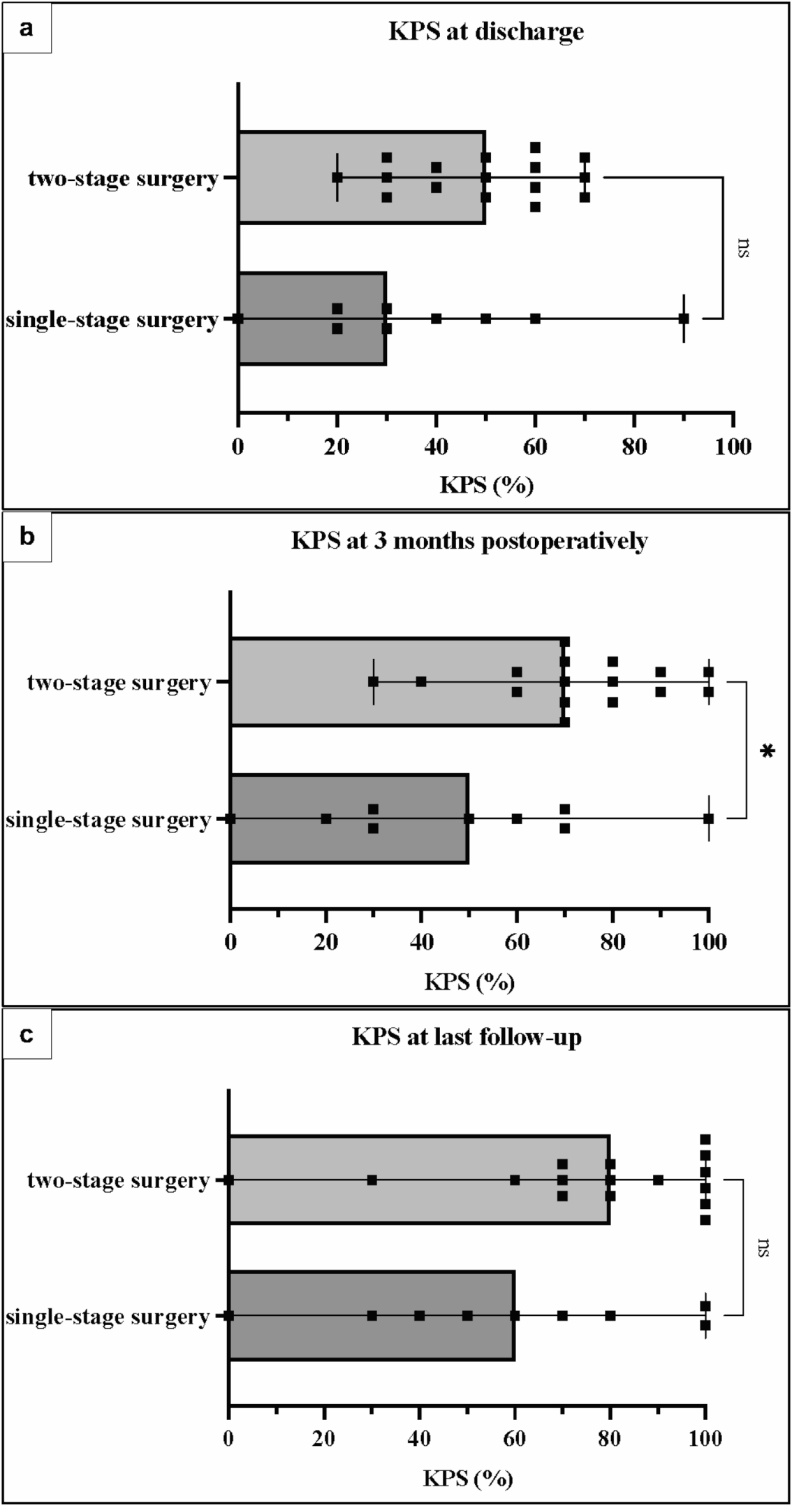
KPS scores: single-stage versus two-stage surgery. The scatter plots show Karnofsky Performance Scale (KPS) scores for each surgical group. Bars indicate median KPS values, the error bars indicate minimum and maximum values. Dots mark each patient’s individual KPS value on the x-axis. Panel a shows KPS at discharge for both groups. Panel b shows KPS at three months postoperatively and Panel c shows KPS at last follow-up for both surgical groups. All patients had meningiomas greater than 50 mm in diameter with large bihemispheric peritumoral edema.

Median KPS at three months postoperatively was significantly higher in Group 2 with KPS 70% (range 30–100%) versus KPS 50% (range 0-100%) in Group 1 (*p* = 0.0204; 95% CI: [4.186, 45.26]). Median KPS at discharge and at the last follow-up were higher in Group 2, but not statistically significant: Median KPS at discharge 50% (range 20–70%) in Group 2 vs. KPS 30% (range 0–90%) in Group 1 (*p* = 0.1829; 95% CI: [-5.874, 29.07]); median KPS at the last follow-up 80% (range 0-100%) in Group 2 vs. KPS 60% (range 0-100%) in Group 1 (*p* = 0.1630; 95% CI: [-7.830, 43.80]) Fig. 3A-C shows patient outcome as measured by KPS at discharge, at three months postoperatively, and at the last follow-up comparing the two surgical groups.

**Fig. 4:**
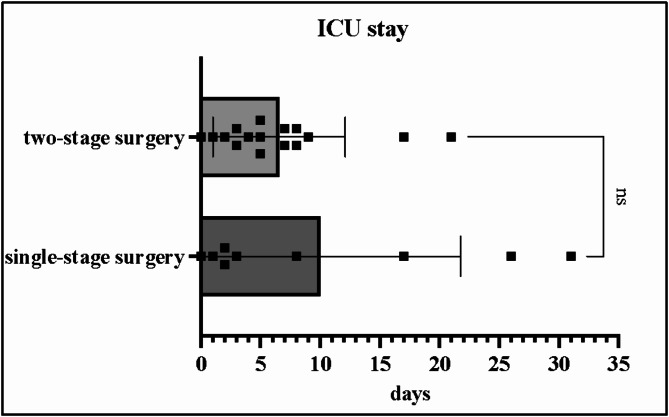
Length of ICU stay. Scatter plot shows mean days in the intensive care unit (ICU) in bars with the error bars marking standard deviation for each surgical group. Dots indicate the length of ICU stay in days for each patient on the x-axis. All patients had meningiomas larger than 50 mm in diameter with large bihemispheric peritumoral edema.

The time from the date of surgery to discharge was shorter for patients in Group 1 without reaching statistical significance (mean 17 [±9] days vs. 24 [±11] days; *p* = 0.1158; 95% CI: [-1.883, 16.05]). However, patients in Group 1 spent more days in the ICU compared to patients in Group 2 (mean 10 [±12] days vs. 6.5 [±6] days; *p* = 0.3284; 95% CI: [-10.56, 3.684]). The length of ICU stay is shown in Fig. 4. In Group 2, 56% of patients had prolonged postoperative ventilation compared to 67% in Group 1 patients (*p* = 0.6274; 95% CI: [-0.5421, 0.3338]).

### Complications

The absolute postoperative complication rate was 67% in Group 1 and 56% in Group 2 (*p* = 0.6274; 95% CI: [-0.5421, 0.3338]), including complications from the second surgical step in Group 2. Of these, the major complication rate was 44% in both surgical groups (*p* = 0.9746; 95% CI: [-0.4530, 0.4391]), including hemorrhage, edema, stroke (due to herniation), hydrocephalus, CSF fistula and sepsis. In Group 1, four patients experienced progressive postoperative edema, with three patients requiring secondary decompressive craniectomy on postoperative days four and ten. All four patients showed reduced vigilance, necessitating prolonged ventilation and invasive ICP monitoring. One of these patients ultimately passed away on postoperative day 30 due to sepsis secondary to ventilator-associated pneumonia.

In Group 2, three patients developed progressive edema between days one and five postoperatively; however, only one of these patients exhibited reduced vigilance and required prolonged ventilation. Additionally, each group had one patient with an epidural hematoma requiring surgical evacuation, occurring on day ten in Group 2 and on day one in Group 1. Minor complications included pneumonia, seizure, pulmonary embolism, and others requiring nonsurgical management such as hyponatremia. Minor complications were more frequent in Group 2 due to five cases of pulmonary embolism (eight complications in six patients; 37.5%) compared to Group 1 (two complications in two patients; 22%; *p* = 0.4530; 95% CI: [-0.2612, 0.5668]).

**Fig. 5:**
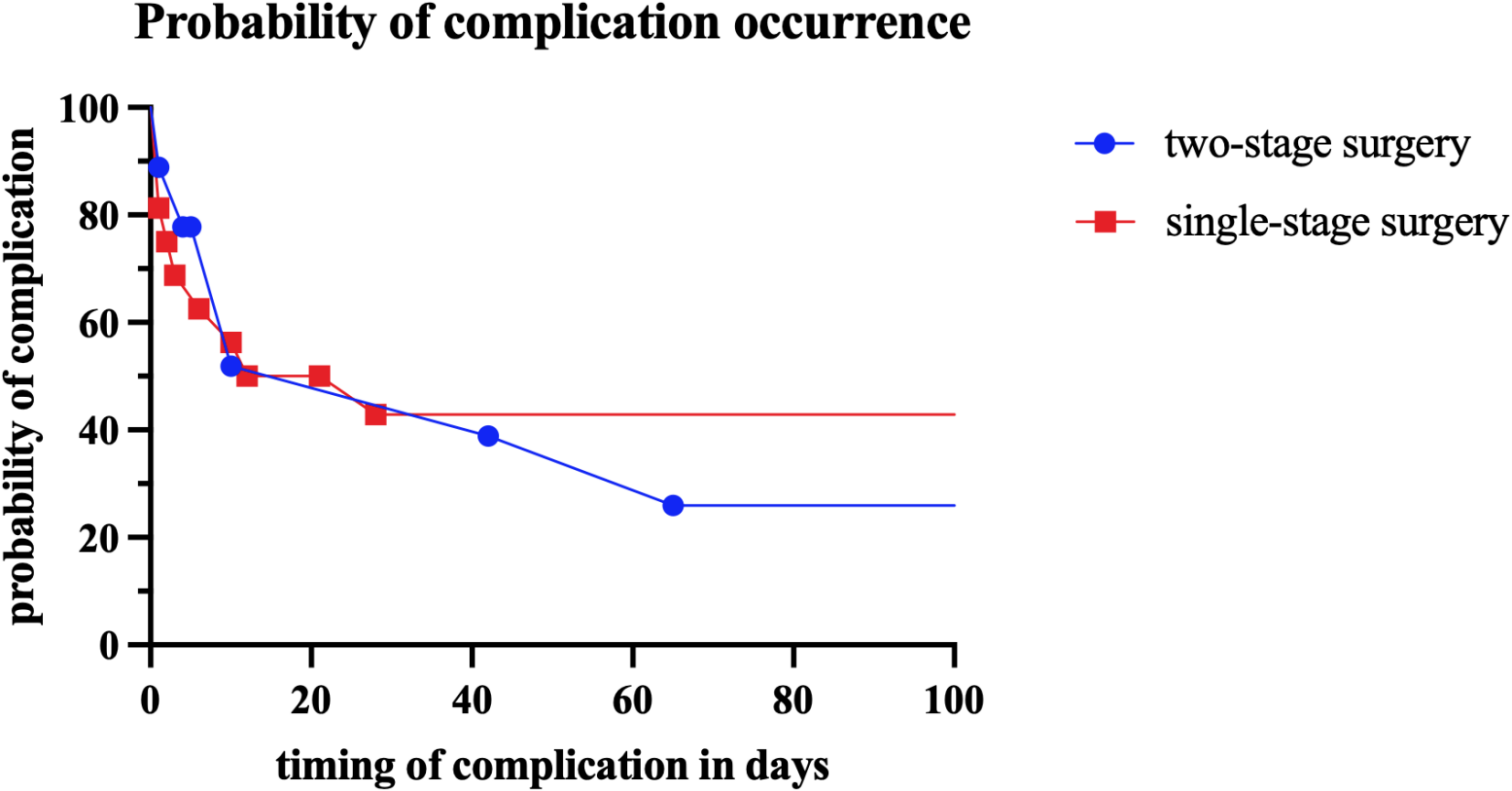
Probability of complication occurrence over time, with the blue line representing Group 1 (single-stage surgery) and the red line representing Group 2 (two-stage surgery). The x-axis indicates time in days, while the y-axis displays the probability as a percentage.

Late postoperative complications were more common in Group 1 patients (44% vs. 19%; *p* = 0.1839; 95% CI: [-0.6449, 0.1310]). In Group 1, two patients developed late-onset hydrocephalus, necessitating shunt implantation on postoperative days 27 and 37, respectively. Additionally, one patient in this group was diagnosed with post-traumatic stress disorder (PTSD) and required long-term professional psychological support. One patient in Group 2 developed an epidural hematoma after cranioplasty requiring surgical evacuation. Table 2 presents the complication profile for each group, while Fig. 5 illustrates the probability of complication occurrence over time.

**Table 2:** Postoperative complications.

	single-stage surgery	two-stage surgery	time point of complication: POD in Group 1 vs. POD in Group 2	*p*-value
total	**16 complications in** **6/9 patients (67%)**	**19 complications in** **9/16 patients (56%)**		0.6274
early postoperative complications	12 complications in 5 patients (56%)	18 complications in 7 patients (44%)		0.3973
major complications	9 complications in 4 patients (44%)	9 complications in 7 patients (44%)		0.9746
hemorrhage	1 *(epidural)*	1 *(epidural)*	1 vs. 10	
CSF fistula	1	3	70 vs. 18–51	
edema	4 *(vigilance reduction*, *2 anisocoria)*	3 *(vigilance reduction)*	1–10 vs. 1–5	
stroke	2 (*herniation)*	0	4–10	
hydrocephalus	0	1	5	
sepsis	1	1	30 vs. 21	
minor complications:	2 complications in 2 patients (22%)	8 complications in 6 patients (37,5%)		0.4530
seizure	0	2	0–12	
pneumonia	1	1	19	
pulmonary emboslism	0	5	6–37	
others	1 (*hyponatremia)*	0	2	
late postoperative complications	4 complications in 4 patients (45%)	2 complications in 3 patients (19%)		0.1839
wound infection	0	1 *(intracerebral abscess)*	28	
late hydrocephalus	2 *(requiring VP-shunt insertion)*	0	27–37	
late epilepsy	1	1	42 vs. 19	
others	1 *(PTSD)*	1 *(EDH following cranioplasty)*	65 vs. 26	

Abbreviation: cerebrospinal fluid (CSF); epidural hematoma (EDH); postoperative day (POD); posttraumatic stress disorder (PTSD); ventriculo-peritoneal (VP).

The mean number of postoperative procedures in both groups was 2.5. Three patients (33%) in Group 1 received a total of 16 interventions after tumor resection, including invasive ICP monitoring, insertion of an external ventricular drain, tracheotomy, evacuation for hemorrhage, VP-shunt insertion and frontobasal covering. All three patients underwent decompressive craniectomy due to progressive brain edema and had more than one postoperative procedure. In Group 2, there were 26 subsequent interventions in 14 patients (88%). This number is high due to the planned autologous cranioplasty as part of the two-stage resection. After subtracting the planned cranioplasties, there were 12 subsequent procedures in five patients (31%). Table 3 provides an overview of the procedures performed after tumor resection.

**Table 3:** Following interventions.

	single-stage surgery	two-stage surgery
total n (% of total)	**16 interventions in** **3/9 patients (33%)**	**26 interventions in** **14/16 patients (88%)**
wound revision	0	2 *(in the same patient;* 12,5%)
frontobasal covering	1 (11%)	3 (19%)
VP shunt implantation	2 (22%)	0
evacuation for hemorrhage	1 (11%)	2 (12,5%)
decompressive craniectomy	3 (33%)	0
tracheotomy	3 (33%)	2 (12,5%)
EVD/ICP-monitoring	4 (44%)	3 (19%)
cranioplasty	2 (22%)	14 (88%)

Abbreviation: external ventricular drain (EVD); intracranial pressure (ICP); ventriculo-peritoneal (VP).

The most common neurological deficit in both groups was psychomotor dysfunction (two patients in Group 1 [22%] vs. seven patients in Group 2 [44%]). The rate of postoperative deficits in both groups was 44% (4/9 patients vs. 7/16 patients) including psychomotor dysfunction, aphasia (*n* = 1 in Group 2), visual impairment (*n* = 1 in Group 2), tetraparesis due to stroke after herniation (*n* = 2 in Group 1) and psychological disorder (*n* = 1 in Group 1).

## Discussion

**Fig. 6:**
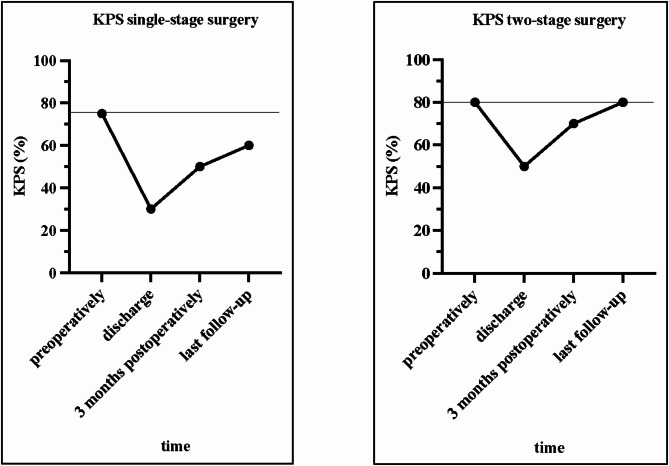
Progression of KPS scores. Compares the progression of the Karnofsky Performance Scale (KPS) score from the preoperative phase to the last follow-up. Median KPS scores at 4 different time points are indicated by dots. The preoperative KPS is indicated by a horizontal line. The left panel shows the KPS evolution for Group 1, while the right panel shows the KPS evolution for Group 2.

The results of this study highlight the high risk and complex complication profile of surgical treatment of anterior midline skull base meningiomas with decompensated peritumoral edema. Two-stage surgery for these high-risk patients demonstrates superior clinical outcomes in terms of KPS three months after surgery compared to a single-stage procedure. In long-term follow-up, patients undergoing two-stage surgery recover to their preoperative clinical status, while patients who undergo single-stage surgery remain at a reduced clinical performance level. Figure 6 compares the evolution of KPS over time in both surgical groups. In addition, the complication profiles for both treatment strategies are similar, even though patients undergoing the two-stage procedure require a definitive secondary intervention for cranioplasty.

Surgery for giant meningiomas arising from the midline of the anterior skull base is associated with a high risk of morbidity and mortality^1,4,6–8,11,18,19^. Cushing reported a mortality rate of 22.7% in the first series of 28 cases of OGM published in 1938^20^ while current reports still describe a perioperative mortality rate of 5%^1,6–8^. The overall complication rates reported in the literature are high, reaching up to 39%^1,4,6–8^, with an increased risk of complications observed in tumors larger than 40 mm (53% vs. 28%) and in those with extensive peritumoral edema (59% vs. 19%)^6^.

Chi et al.^9^ classified peritumoral edema in anterior midline skull base meningiomas into four subtypes based on radiographic appearance: (A) no edema; (B) mild edema, limited to the rectus gyrus; (C) moderate edema, extending beyond the rectus gyrus and (D) severe edema, extensive bilateral edema. All patients included in our study showed the highest category D edema formation. Several authors have reported an association between peritumoral brain edema and increased morbidity and mortality in meningioma patients^2,7,8,21^.

Mukherjee et al.^6^ described a characteristic radiographic pattern of edema extending posteriorly within the external capsule, which is associated with a higher complication rate. All patients included in our series presented with the so-called “sabre-tooth” sign (Fig. 1, white arrow), consequently indicating a higher peri- and postoperative risk of complications. 9% of patients in Mukherjee’s series of 33 patients developed severe postoperative progression of cerebral edema, requiring surgical decompression in all cases and even leading to death in one case^6^. These findings underscore the fact that all patients included in the study presented here were high-risk patients, which accounts for the elevated complication rate observed.

The major complication rate in our series was slightly higher than reported in other series (44% vs. 11–39%^6–10^). While these rates may seem high, they are not unusual for lesions of this nature, given the challenges posed by neurovascular conflicts and narrow surgical corridors. For example, opting not to open the frontal sinus during surgery for large lesions with extensive edema can result in a suboptimal surgical corridor due to the bony overhang, leading to increased brain retraction and potential complications such as frontal lobe contusions.

Additionally, reported complication rates often vary due to differences in inclusion criteria and definitions of complications. We defined all events requiring subsequent treatment (oral or intravenous medication, intervention, surgery, ICU observation) as complications.

Despite the detailed analysis of adverse events, two-stage surgery did not lead to an increased overall complication rate, even with the inclusion of mandatory cranioplasty. Complications associated with cranioplasty, such as infection, implant failure, and bone resorption, are well-documented in the literature^14,15^ and represent a significant consideration in the two-stage surgical management of large skull base meningiomas. Notably, cranioplasty carries a substantial complication risk of 20–45%^13,14^. Mandatory cranioplasty is also reflected in the high number of additional postoperative interventions in patients undergoing two-stage surgery. However, after subtracting cranioplasty, additional postoperative interventions were significantly reduced in patients undergoing two-stage surgery, explaining the comparable overall complication profile for both treatment strategies.

The prolonged hospital stay in Group 2 is attributed to the two surgical steps, which were performed during the same hospital stay if the patient recovered quickly. Patients in Group 1 were often transferred directly to a rehabilitation facility, leading to shorter hospital stays. The extended ICU stay and prolonged postoperative ventilation of Group 1 patients suggests more intensive treatment within the ICU for these patients. This contributes to increased hospital costs, as ICU treatment and monitoring are highly expensive. Nonetheless, for patients in Group 2, a second surgery is mandatory, which further raises the overall costs of hospitalization. However, in some cases, patients who undergo two-stage surgery are no longer observed in the ICU at our department. If prolonged ventilation is not required, they are monitored in the recovery room for several hours before being transferred to the general ward. This approach helps conserve valuable ICU resources for more critically ill patients^22^.

The observed clinical benefit of two-stage surgery may be attributed to a reduction in complications that lead to severe and persistent clinical dysfunction, such as stroke, secondary hydrocephalus, and prolonged ICP decompensation requiring secondary or third-line ICP treatments. However, given that the treatment periods differ between the groups (Group 1: 2002–2016; Group 2: 2012–2022), advancements in surgical techniques and diagnostic tools over time may have contributed to improved patient outcomes. For instance, surgical approaches continue to be a topic of research, with findings suggesting a slight outcome benefit associated with the subfrontal approach versus interhemispheric approach^23^. Nevertheless, surgery for these tumors remains invasive and aggressive, regardless of advancements in technology or techniques, as reflected by the high complication rates. Given these elevated risks, a two-stage approach may only be preferable for giant meningiomas (≥ 5 cm) located in the anterior midline skull base, typically olfactory groove meningiomas, accompanied by extensive, bihemispheric edema (classified as category D according to Chi et al.^9^ or identified by the “sabre-tooth” sign per Mukherjee et al.^6^). In our department, the decision between single- and two-stage procedures is based on tumor size and edema extent, with a two-stage resection routinely performed when both criteria (tumor size ≥ 5 cm and extensive edema) are met.

Limitations of the current study include the small sample size, the single-center study design, and the retrospective analysis approach. We acknowledge that the limited sample size, particularly in our single-stage surgery group, restrict statistical power and generalizability. This is a consequence of the highly selective nature of the patient cohort considered for two-stage surgery. Furthermore, surgical techniques and practices have evolved over time, which may introduce challenges when comparing historical and modern groups, although advancements in surgical technology for these tumor types have not necessarily resulted in major advantages. Our department no longer performs single-stage surgery on patients with giant olfactory groove meningiomas and extensive bihemispheric edema given the demonstrated benefits of the two-stage approach. Consequently, increasing the sample size of the single-stage group within this study is not feasible. A randomized trial in these selected high-risk patient population presents challenges due to the small number of eligible patients, ethical concerns regarding the randomization process, and the urgent need for treatment. However, multicenter analyses could validate these findings across larger cohorts. Future studies should also include cognitive or quality-of-life measures, especially given the neuropsychological impacts of these tumors and their treatment.

## Conclusion

In conclusion, two-stage surgery for large anterior midline skull base meningiomas with decompensated peritumoral edema is associated with statistically significant improved clinical outcomes at three months post-surgery and non-significant improvements at discharge and last follow-up compared to a historical single-stage surgery control group. These findings, derived from a highly selected patient population in a non-randomized and single-center study, suggest that two-stage surgery does not increase the complication profile. To establish stronger scientific evidence on the optimal surgical approach for these high-risk patients, a multi-center prospective study with a larger patient cohort is highly recommended.

## Data Availability

The datasets used and/or analysed during the current study available from the corresponding author on reasonable request.

## Abbreviations

ICP: intracranial pressure
ICU: intensive care unit
KPS: Karnofsky Performance Scale
MRI: magnetic resonance imaging
OGM: olfactory groove meningioma
PSM: planum sphenoidale meningioma
TSM: tuberculum sellae meningioma

## References

[1] Hentschel, S. J. Olfactory groove meningiomas. *Neurosurg. Focus*. **14**, 5 (2003). 10.3171/foc.2003.14.6.4 15669789

[2] Vignes, J. R., Sesay, M., Rezajooi, K., Gimbert, E. & Liguoro, D. Peritumoral edema and prognosis in intracranial meningioma surgery. *J. Clin. Neurosci.* **15**, 764–768 (2008). 18406142 10.1016/j.jocn.2007.06.001

[3] Department of Neurosurgery, University of Health Sciences, Gulhane Education and Research Hospital, Ankara, Turkey, Yasar, S. & Kirik, A. Department of Neurosurgery, University of Health Sciences, Gulhane Education and Research Hospital, Ankara, Turkey (2021) Surgical Management of Giant Intracranial Meningiomas. Eurasian J Med 53:73–78. 10.5152/eurasianjmed.2021.20155 PMC8184033 34177286

[4] El Gindi, S. Olfactory groove meningioma: surgical techniques and pitfalls. *Surg. Neurol.* **54**, 415–417 (2000). 11240167 10.1016/s0090-3019(00)00346-3

[5] Zada, G., Başkaya, M. K. & Shah, M. V. Introduction: surgical management of skull base meningiomas. *Neurosurg. Focus*. **43**, Intro (2017). 28967307 10.3171/2017.10.FocusVid.Intro

[6] Mukherjee, S. et al. Resection of olfactory groove meningioma – a review of complications and prognostic factors. *Br. J. Neurosurg.* **29**, 685–692 (2015). 26174632 10.3109/02688697.2015.1054348

[7] de Aguiar, P. H. P. et al. Olfactory groove meningiomas: approaches and complications. *J. Clin. Neurosci.* **16**, 1168–1173 (2009). 19577476 10.1016/j.jocn.2008.12.013

[8] Nakamura, M., Struck, M., Roser, F., Vorkapic, P. & Samii, M. OLFACTORY GROOVE MENINGIOMAS: CLINICAL OUTCOME AND RECURRENCE RATES AFTER TUMOR REMOVAL THROUGH THE FRONTOLATERAL AND BIFRONTAL APPROACH. *Neurosurgery* **60**, 844–852 (2007). 17460519 10.1227/01.NEU.0000255453.20602.80

[9] Chi, J. H., Parsa, A. T., Berger, M. S., Kunwar, S. & McDermott, M. W. Extended bifrontal craniotomy for midline anterior Fossa meningiomas:minimization of Retraction-Related edema and surgical outcomes. *Oper. Neurosurg.* **59**, ONS-426–ONS-434 (2006). 10.1227/01.NEU.0000223508.60923.91 17041513

[10] Pallini, R. et al. Olfactory groove meningioma: report of 99 cases surgically treated at the Catholic university school of medicine. *Rome World Neurosurg.* **83**, 219–231e3 (2015). 25464274 10.1016/j.wneu.2014.11.001

[11] Sankhla, S. K., Jayashankar, N., Khan, M. A. & Khan, G. M. Surgical Management of Tuberculum Sellae Meningioma: Our Experience and Review of the Literature. Neurol India. (2021). Nov-Dec;69(6):1592–1600 10.4103/0028-3886.333529. PMID: 34979648. 10.4103/0028-3886.333529 34979648

[12] Marquardt, G. et al. Two-step staged resection of giant olfactory groove meningiomas. *Acta Neurochir. (Wien)*. **163**, 3425–3431 (2021). 34373942 10.1007/s00701-021-04910-3 PMC8599346

[13] Shepetovsky, D., Mezzini, G. & Magrassi, L. Complications of cranioplasty in relationship to traumatic brain injury: a systematic review and meta-analysis. *Neurosurg. Rev.* **44**, 3125–3142 (2021). 33686551 10.1007/s10143-021-01511-7 PMC8592959

[14] Goedemans, T. et al. Complications in cranioplasty after decompressive craniectomy: timing of the intervention. *J. Neurol.* **267**, 1312–1320 (2020). 31953606 10.1007/s00415-020-09695-6 PMC7184041

[15] Hutchinson, P. J. et al. Decompressive craniectomy versus craniotomy for acute subdural hematoma. *N Engl. J. Med.* **388**, 2219–2229 (2023). 37092792 10.1056/NEJMoa2214172

[16] Baumgarten, P. et al. Early and late postoperative seizures in meningioma patients and prediction by a recent scoring system. *Cancers* **13**, 450 (2021). 33504023 10.3390/cancers13030450 PMC7865990

[17] Wirsching, H-G. et al. Predicting outcome of epilepsy after meningioma resection. *Neuro-Oncol* **18**, 1002–1010 (2016). 26683139 10.1093/neuonc/nov303 PMC4896539

[18] Aftahy, A. K. et al. Midline meningiomas of the anterior skull base: surgical outcomes and a Decision-Making algorithm for classic skull base approaches. *Cancers* **12**, 3243 (2020). 33153110 10.3390/cancers12113243 PMC7692292

[19] Tuna, H., Bozkurt, M., Ayten, M., Erdogan, A. & Deda, H. Olfactory groove meningiomas. *J. Clin. Neurosci.* **12**, 664–668 (2005). 16109489 10.1016/j.jocn.2005.05.002

[20] Cushing, H. & Eisenhardt, L. Meningiomas. Their Classification, Regional Behaviour, Life History, and Surgical End Results. 1938;27(2):185. Springfield, II: Charles C. Thomas (1938).

[21] Gurkanlar, D., Er, U., Sanlı, M., Özkan, M. & Sekerci, Z. Peritumoral brain edema in intracranial meningiomas. *J. Clin. Neurosci.* **12**, 750–753 (2005). 16165364 10.1016/j.jocn.2004.09.029

[22] Qasem, L-E. et al. Implementation of the no ICU – Unless approach in postoperative neurosurgical management in times of COVID-19. *Neurosurg. Rev.* **45**, 3437–3446 (2022). 36074279 10.1007/s10143-022-01851-y PMC9452872

[23] Abou-Al-Shaar, H., Patel, K. P., Mallela, A. N. & Sekula, R. F. Lateral supraorbital approach for resection of large and giant olfactory groove meningiomas: a single center experience. *Br. J. Neurosurg.* **37**, 90–96 (2023). 36053047 10.1080/02688697.2022.2117273

